# Unconscious cognition without post hoc selection artifacts: From selective analysis to functional dissociations

**DOI:** 10.3758/s13428-025-02926-6

**Published:** 2026-01-16

**Authors:** Thomas Schmidt, Maximilian P. Wolkersdorfer, Xin Ying Lee, Omar Jubran

**Affiliations:** 1 Visual Attention and Awareness Laboratory, University of Kaiserslautern-Landau (RPTU), Erwin-Schrödinger-Str. Geb. 57, 67663, Kaiserslautern, Germany; 2 Department of Cognitive and Developmental Psychology, University of Kaiserslautern-Landau (RPTU), Erwin-Schrödinger-Str. Geb. 57, 67663 Kaiserslautern, Germany

**Keywords:** Unconscious Cognition, Post-Hoc Sorting, Functional Dissociations

## Abstract

One of the most popular approaches to unconscious cognition is the technique of “post hoc selection”: Priming effects and visibility ratings are measured in multitasks on the same trial, and only trials with the lowest visibility ratings are selected for analysis of (presumably unconscious) priming effects. In the past, the technique has been criticized for creating statistical artifacts and capitalizing on chance. Here, we argue that post hoc selection constitutes a sampling fallacy, confusing sensitivity and response bias, wrongly ascribing unconscious processing to stimulus conditions that may be far from indiscriminable. In response to a high-profile “best practice” paper by Stockart et al. (2025) that condones the technique, we use standard signal detection theory to show that post hoc selection only isolates trials with neutral response bias, irrespective of actual sensitivity, and thus fails to isolate trials where the critical stimulus is “unconscious”. Our own data demonstrate that zero-visibility ratings are consistent with uncomfortably high levels of sensitivity. As an alternative to post hoc selection, we advocate the study of functional dissociations, where direct (*D*) and indirect (*I*) measures are conceptualized as spanning a two-dimensional *D-I* space wherein simple, sensitivity, and double dissociations appear as distinct curve patterns. While Stockart et al.’s recommendations cover only a single line of that space where *D* is close to zero, functional dissociations can utilize the entire space. This circumvents requirements like null visibility and exhaustive reliability, allows for dissociations among different measures of awareness, and supports the planful measurement of functional relationships between direct and indirect measures.

## Dissociation curves: A journey through *D-I* space

In a high-profile collaboration of 32 authors, Stockart et al. (2025)^1^ present the outcome of a number of discussion meetings on “best practices” in the methodology of unconscious cognition research. The result may be viewed as a tactical compromise between a majority recommending a practice called *post hoc selection* and a minority group strongly criticizing this practice on mathematical grounds. Here we will argue that even though some of the authors are leading experts in the field and we agree with many of their recommendations, the compromise on post hoc selection in particular is not viable and that the practice needs to be abandoned because it fails to isolate stimulus conditions where perceptual sensitivity is low. At the same time, we want to point out that there are many attractive alternatives to post hoc selection that hold great promise for advancing the field. Therefore, this paper should be viewed not only as a critique of post hoc selection but also as an invitation to the fascinating field of *functional dissociations* in the study of unconscious cognition.

**Fig. 1:**
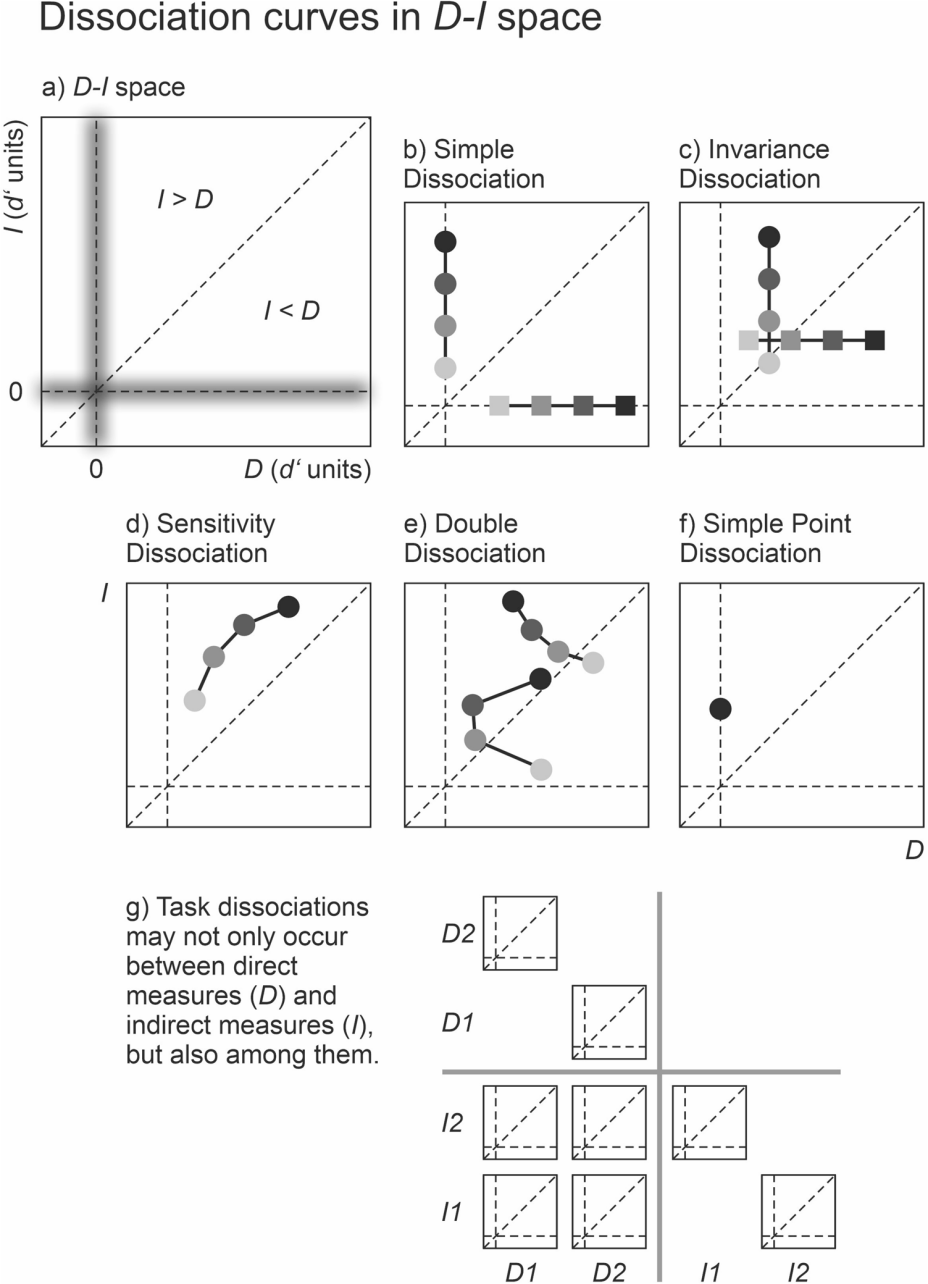
Dissociation curves between a direct and an indirect measure. **a)** In a *D-I* space, indirect effects are plotted against direct effects after conversion to a shared metric (here, *d’*). Fuzzy lines at effect sizes of *D* = 0 and *I* = 0 symbolize the statistical problem of determining whether effects differ from null performance. **b)** Simple dissociations show variation in one effect while the other effect is zero. **c)** Invariance dissociations describe the more general case where the invariant effect is constant at some fixed value, not necessarily zero. **d)** Sensitivity dissociations show one effect to be clearly larger than the other. **e)** Double dissociations show that an experimental manipulation has opposite effects on both measures: The two examples show increasing indirect effects under either decreasing or U-shaped direct effects (e.g., “type B masking”). **f)** Of all those possibilities, Stockart et al.’s paper focuses on a particular special case of **b)**, the simple point dissociation. **g)** Task dissociations may occur not only between but also among direct and indirect measures, each coming with its own decision space and criterion content (Kahneman, 1968; T. Schmidt & Biafora, 2024). Here, all possible spaces between two direct measures (*D1*, *D2*) and two indirect measures (*I1*, *I2*) are plotted. – In all plots, shading of the data points indicates some experimental variation that leads to increasing indirect effects, like increasing prime-target interval or prime intensity. However, dissociation patterns are defined solely by their shape in *D-I* space, not their directionality

What are functional dissociations, and what is special about them? Stockart et al.‘s paper is anchored in the *dissociation paradigm*, which consists of comparing a direct measure (*D*) of awareness with an indirect measure (*I*) that implies that the critical stimulus or feature was cognitively processed (Reingold & Merikle, 1988; see also Cheesman & Merikle, 1984, 1986; Erdelyi, 1986; Snodgrass et al., 2004). For instance, the indirect measure may be a priming effect in response times generated by a *critical feature* of the prime (e.g., its color), and the direct measure may be some measure of awareness of that critical feature (e.g., objective discrimination performance or subjective visibility ratings). To appreciate the full range of possibilities of the dissociation paradigm, consider Fig. 1a. If we plot performance in the indirect measure against performance in the direct measure, we span a *D-I space* (T. Schmidt, 2000; T. Schmidt & Vorberg, 2006). There is a horizontal line where the indirect effect is zero and a vertical line where the direct effect is zero. If we express *D* and *I* in the same metric (T. Schmidt & Vorberg, 2006; e.g., the effect-size metric *d’* of signal detection theory [SDT]), the main diagonal indicates cases where direct and indirect effects are identical in magnitude. For cases falling above the diagonal, the indirect effect is larger than the direct one, and vice versa below the diagonal. Different types of dissociations appear as distinct patterns when plotted in *D-I* space. We are especially interested in data patterns where each of the two measures appears as a *function of a parametric variable*, for example, a variation in contrast or in stimulus-onset asynchrony (SOA) between prime and target. When the parametric courses of the two functions lead to interesting discrepancies, we speak of *functional dissociations*. They appear as *dissociation curves* in *D-I* space.

Figure 1 shows various possibilities of dissociation curves between direct and indirect measures (T. Schmidt & Biafora, 2024; T. Schmidt & Vorberg, 2006). *Simple dissociations* (Fig. 1b, circles) occur when the critical feature induces an indirect effect while a direct measure of awareness for that feature shows zero performance. Examples for this data pattern can be seen in Vorberg et al. (2003) and F. Schmidt & T. Schmidt (2010). The overwhelming majority of studies in unconscious cognition are based on such simple dissociations. Note that the reverse pattern (variation in awareness without any indirect effect; Fig. 1b, squares) also constitutes a simple dissociation and can also be of theoretical interest.

Simple dissociations are special cases of *invariance dissociations*, which are not confined to null effects. Generally, variation in *I* at an invariant level of *D* (Fig. 1c, circles) indicates that changes in the direct measure are *not necessary* for changes in the indirect measure. Correspondingly, variation in *D* at an invariant level of *I* (Fig. 1c, squares) shows that changes in the direct measure are *not sufficient* for producing changes in the indirect measure. The Fehrer–Raab effect (Fehrer & Biederman, 1962; Fehrer & Raab, 1962) is a classic example of an invariance dissociation where response times to a masked stimulus remain constant under large variations in awareness (metacontrast masking). Similarly, Lamy et al. (2017) argue that response priming remains unaffected when visual awareness for a masked prime is enhanced by previous presentation.

Arguments have also been based on a magnitude comparison between direct and indirect measures. Reingold and Merikle (1988) introduced the idea of a *sensitivity dissociation* where the indirect effect is shown to be clearly larger than the direct one. Sensitivity dissociations imply data points above the diagonal of the *D-I* plot (Fig. 1d). Meyen et al. (2022; see also Zerweck et al., 2021) argue that dissociations are only convincing if the indirect effect is clearly larger than the direct one. Sensitivity dissociations (unlike other dissociation types) require equal scaling of the two measures and the measurement-theoretical assumption that the direct measure responds at least as sensitively to conscious information as the indirect measure (T. Schmidt & Vorberg, 2006). Several techniques have been proposed for expressing direct and indirect effects on the same scale. Response time effects are often converted into *d’* units by performing a median split on the distribution and then cross-tabulating fast and slow responses with prime-target congruency to generate hits and false alarms (Meyen et al., 2022; T. Schmidt, 2002; Zerweck et al., 2021). This type of analysis provides counts of how many “fast” and “slow” responses occur in congruent as compared to incongruent trials. However, this down-transformation of RT to a dichotomous measure discards valuable information. Instead of defining *d’* via the counts of hits and false alarms, one can also work from the idea that *d’* is conceptually an effect-size measure defined as the distance between two standardized distributions, which in the case of RTs are observable. T. Schmidt and Vorberg (2006) recommend calculating *d’* directly from the distance between the RT distributions (standardized by their pooled, winsorized standard deviation). That method obviously requires access to the raw data. Meyen et al. (2024) propose a method for converting published *t* values of RT effects into *d’* values.

Simple and invariance dissociations can be quite convincing when the measurement is highly accurate and the invariance is stable over many conditions. However, they share a fundamental problem: We can only infer invariance of awareness from invariance of the direct measure if we can assume that this measure is both exhaustively valid (T. Schmidt & Biafora, 2024) and exhaustively reliable (Reingold & Merikle, 1988; formal definitions in T. Schmidt & Biafora, 2024). *Exhaustive validity* means that the direct measure must capture all information sources theoretically relevant for the direct task. In particular, the direct measure is required to capture awareness of the *critical feature* (the stimulus property that drives the indirect effect; e.g., prime color in a color priming task). *Exhaustive reliability* means that the direct measure is a strictly (not only weakly) monotonic function of conscious information. To illustrate this critical concept, consider a mechanical barometer supposed to measure air pressure. A *strictly monotonic* barometer is one that never gets stuck and will respond to any change in air pressure, no matter how small. In contrast, a *weakly monotonic* barometer is allowed to sometimes get stuck and require a knock on the glass to reveal movement of the needle. It may even be broken and never move at all – the only requirement for weak monotonicity is that it doesn’t systematically respond in the wrong direction (T. Schmidt & Vorberg, 2006). Applied to the measurement of visual awareness, weak monotonicity is a benign assumption that needs to be placed on any regular measurement process, whereas strict monotonicity is a severe assumption because it requires the direct measure to pick up any minuscule change in awareness (Malejka et al., 2021; Reingold & Merikle, 1988). Of course, no psychometric measure can plausibly be assumed to be strictly monotonic, i.e., have unlimited sensitivity to change.

Apart from this measurement-theoretical problem, a simple dissociation is very difficult to establish to begin with because no procedure exists that ensures chance-level performance at the group level of observers, at least not in visual masking. Although there are lawful relationships between stimulus variables and the typical shapes of masking functions (Breitmeyer & Öğmen, 2006; Kahneman, 1968), masking functions still vary quantitatively and qualitatively from person to person. Even with the same set of stimuli, individual observers’ performance may be at chance, at ceiling, or show increasing, decreasing, or U-shaped masking functions. As fascinating as these individual variations often are, they are problematic when hunting for chance-level effects at the group level: For the group-averaged masking function to be close to chance, the great majority of individual observers must be close to chance as well, and that simply does not occur so easily. Staircase or threshold procedures as proposed by Shanks (2017) may sometimes help with idiosyncratic masking functions, but more often than not fail to compensate for the qualitative differences in data patterns. This has a very important and underestimated consequence for the entire classical dissociation logic: *Simple dissociations depend on sheer luck.* Only once in a while do we have the good fortune of finding a group-averaged masking function close to zero with small error bars and good agreement between observers. Then we have a “proof of concept” that, say, response priming can occur without prime discrimination (for some convincing examples, see Vorberg et al., 2003, Exp. 1, or F. Schmidt & T. Schmidt, 2010). However, as soon as we repeat the experiment, we will have a new group of observers with their idiosyncratic masking functions. Fortunately, we can measure each individual observer with high precision by using massively repeated measures, so our problem is actually not measurement noise (failure to replicate at the individual level) but real idiosyncrasy (high replicability within observers, but variation of data patterns between observers). The dependence of the simple dissociation paradigm on blind luck has certainly been a major driving force in developing methods that promise to identify “unconscious” trials irrespective of the concrete shape of the masking function.^2^

However, there actually is a data pattern that does not require invisible stimuli at all: *double dissociation*. A double dissociation is demonstrated when the two measures show opposite courses under parametric variation of an independent variable. For instance, this is the case when priming effects increase despite decreasing performance in prime discrimination or subjective visibility. When plotted in *D-I* space, double dissociation leads to a dissociation curve that slopes downward somewhere along its course (Fig. 1e). The measurement-theoretical foundations have been worked out in detail (T. Schmidt & Vorberg, 2006; cf. Ashby & Bamber, 2022; Dunn & Kirsner, 1988; Merikle & Joordens, 1997, for closely related concepts). Most importantly, double dissociations need no exhaustiveness assumptions, and they do not require zero visibility of the critical feature – indeed they disintegrate to simple dissociations when all their data points come to fall on the *D* = 0 line. All they require are some assumptions of weak monotonicity, and even those only have to hold in the long run (on the level of expected values, see below). In research on unconscious perception, double dissociations have primarily been observed for response priming under metacontrast masking. Under suitable stimulus conditions, metacontrast can lead to masking functions that are decreasing or U-shaped when prime-mask SOA is varied, while priming effects increase with SOA (Biafora & T. Schmidt, 2020, 2022; Koster et al., 2020; Mattler, 2003; Vorberg et al., 2003; Yang & Rouder, 2025).

Double dissociations are not confined to metacontrast or even to masked priming. However, finding them sometimes requires a bit of creativity. For instance, Spering et al. (2011; Spering & Carrasco, 2015) studied drifting grating patterns to dissociate conscious movement perception from smooth pursuit eye movements. They presented two grating patterns, one horizontal and one vertical, dichoptically to the two eyes. While the resulting motion perception simply was the vectorial sum of both patterns, adaptation studies showed that pursuit movements followed the component patterns instead. Similarly, T. Schmidt et al. (2010) employed the corrugated plaid illusion (Adelson, 1993), a lightness illusion that affected the apparent lightness of a prime stimulus but left the target intact. They showed that response priming effects only depended on the local stimulus contrast of the primes, not on their perceived lightness: One prime could appear lighter than another (in a direct matching task), but activate responses as if it were the darker one. The result was a double dissociation between color appearance and response activation, showing that response priming does not depend on perceived surface color (T. Schmidt, 2021).

We have recently developed the experimental technique of “induced dissociations” that can help bend masking functions into a desired shape without affecting response activation from the prime (Biafora & T. Schmidt, 2020). This technique involves a combination of two independent variables: a *driver variable* that modulates the indirect effect, and a *bender variable* that modulates the direct effect but ideally does not affect the indirect one. For instance, response priming is strongly modulated by prime-mask SOA, which makes SOA a suitable driver variable for priming. Mask contrast, on the other hand, can be used as a bender variable because it modulates metacontrast masking without affecting response priming (at least if the stimuli are constructed in the right way; see Biafora & T. Schmidt, Exp. 3). The trick of the design is in the combination of the driver and the bender variables. If mask contrast is simply high or low throughout all SOA conditions, so that the bender variable is constant, we simply compare strong and weak masking, as many studies do. However, if mask contrast systematically increases or decreases as a function of SOA, it is sometimes possible to create two masking functions that proceed in opposite directions, while response priming effects continue to increase. With this technique, we were able to create, for the first time, double dissociations between response priming by color and color discrimination performance.

Stockart et al. (2025) do not seem to be aware of the full range of possibilities in functional dissociation patterns. Most of the time, they discuss their methods by means of a simple priming paradigm (based on Dehaene et al., 1998) that generates only a single data point on the single line in *D-I* space where *D* is exactly zero. We call this scenario a *simple point dissociation* (Fig. 1f); it is a special case of a simple, invariance, or sensitivity dissociation crunched into a single coordinate. Many of the problems with Stockart et al.’s recommendations arise from the attempt to draw spurious information from such a single data point – specifically, from the noise in the data point.

We now look at what we consider the most important of Stockart et al.’s recommendations (Table 1). We focus on the list presented in their Fig. 2, using our own thematic sorting (some points appear more than once, some not at all). We do not use Stockart et al.’s original numeration because it has changed between different versions of the manuscript. We urge readers to read their text in detail to appreciate the full range of pro and con arguments the authors provide. Note that most of their recommendations are based on a solid majority of votes, and only a few recommendations met with some dissent.

**Table 1:** List of recommendations as it appears in Fig. 2 of Stockart et al. (2025). “Agreement” refers to the proportion of coauthors of that paper who agreed with the statement

Statement	Agreement
Use both subjective and objective direct measures.	94 %
Objective direct measure: Use a forced-choice discrimination task on the feature of interest.	91 %
Subjective awareness measure: A preference towards the PAS.	78 %
Collect all measures on a trial-by-trial basis.	94 %
Add catch trials in which no stimulus is presented.	91 %
Ensure adequate precision of the processing and awareness measures.	100 %
When using the PAS, trials rated as “brief glimpse” should not be classified as “unaware”.	85 %
Examine the possible effect of misclassification due to measurement error.	97 %
Precisely define what is meant by “unconscious processing”.	100 %
Justify the chosen measures and provide conditional interpretations.	97 %

**Fig. 2:**
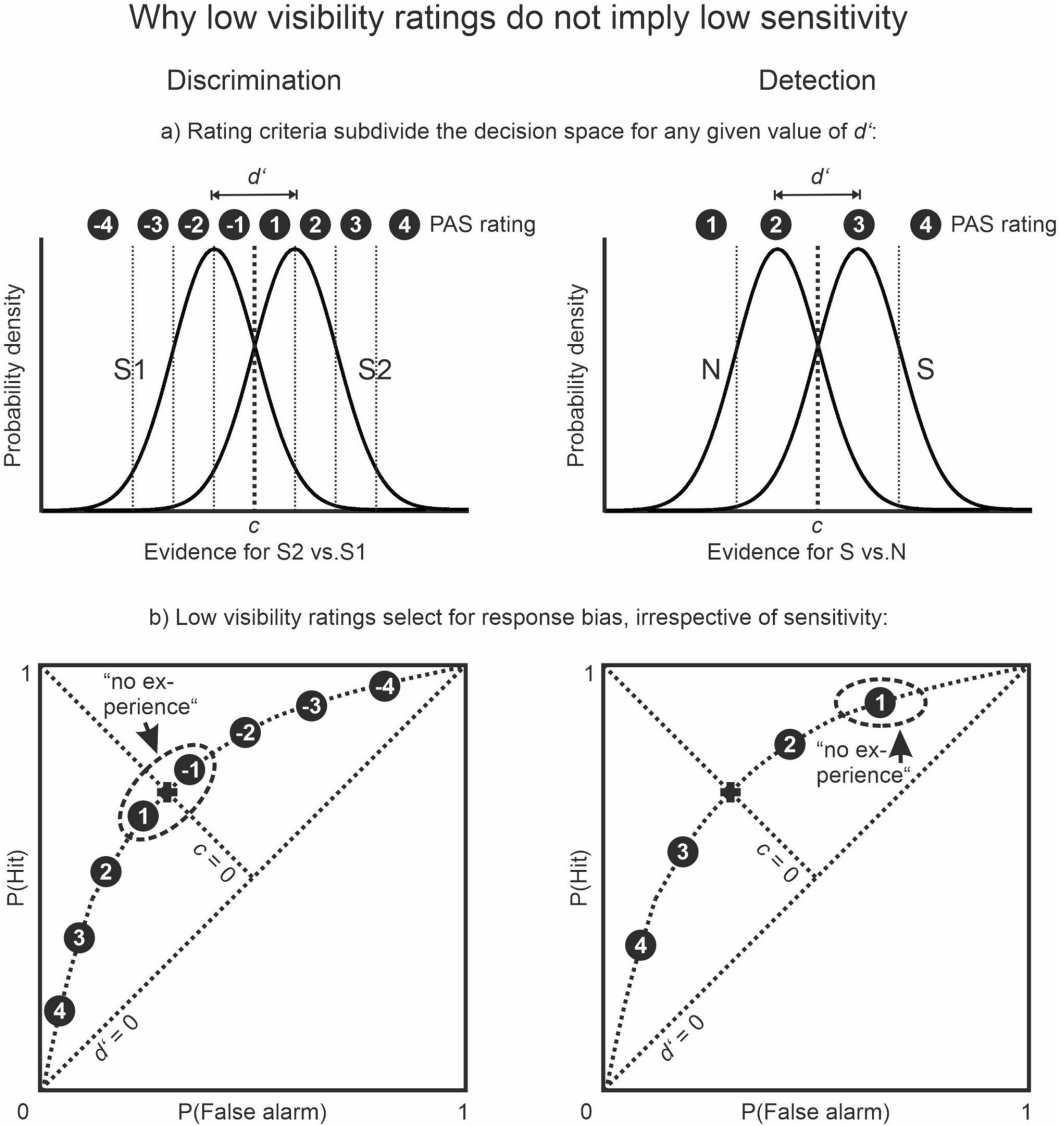
Signal detection models for joint PAS ratings and objective discrimination or detection judgments in a single experimental condition. *Left*: discrimination task. *Right*: detection task. **a)** Internal evidence for the two stimulus alternatives (Stimulus 1 versus Stimulus 2, or Stimulus versus Noise). Without loss of generality, both stimuli are assumed to lead to identical standard normal distributions along the decision axis, so that the sensitivity index d’ simply corresponds to the standardized distance between the distributions. The response criterion (c, heavy vertical line) is assumed to be optimal here, minimizing the number of classification errors. In discrimination, hits are arbitrarily defined as “S2” decisions to S2 stimuli, false alarms as “S2” decisions to S1 stimuli. In detection, hits are defined as “S” decisions to S stimuli, false alarms as “S” responses to N stimuli. PAS visibility ratings range from 1 to 4, with a negative sign attached whenever the critical stimulus is identified as S1 in the concurrent discrimination task. Rating categories are separated by additional response criteria (light vertical lines). **b)** ROC curves plot cumulative rates for hits versus false alarms for PAS categories in ascending order. On the curve, the marking “4” stands for hits and false alarms in all rating categories ≥ 4, “3” for all rating categories ≥ 3, and so on. Major diagonal: marks *d’* = 0 where hit rate equals false alarm rate. Minor diagonal: marks neutral response criterion *c* = 0. The *dotted curve* marks an isosensitivity curve where all points have the same sensitivity *d’* but may differ in response criterion. The *plus sign* marks the spot of neutral response bias for an ideal observer. The lowest ratings (±1) are marked with the PAS label “no experience” (Ramsøy & Overgaard, 2004). Note that they are part of the same ROC as all the other rating categories and thus indicate the same sensitivity

### Choice of measures and measure-specific interpretation

*“Objective direct measure: Use a forced choice discrimination task on the feature of interest.”*

*“Subjective awareness measure: A preference towards the PAS.”*

*“Precisely define what is meant by ‘unconscious processing’.”*

*“Justify the chosen measures and provide conditional interpretations.”*

*(from *Stockart et al., 2025*, *Fig. 2*)*

We start with some general considerations about the measurement of visual awareness.

Any dissociation between one direct and one indirect measure is only a special case of a larger space of *task dissociations* (Fig. 1g). This approach acknowledges that there are not only dissociations *between* direct and indirect measures, but also *among* them (Koster et al., 2020; T. Schmidt & Biafora, 2024). Direct as well as indirect tasks differ in the *decision spaces* they require, which predict distinct patterns of sensitivity and response biases when, for example, detection, discrimination, and same-different tasks are compared (Hautus et al., 2022). Many details of task performance will depend on the particular geometry of the decision space (Hellmann et al., 2023; Locke et al., 2022; Malejka & Bröder, 2019; Rausch et al., 2021; Zehetleitner & Rausch, 2013) and on the way response criteria are placed for different types of measures (Macmillan, 1986; Maniscalco & Lau, 2012, 2014; Maniscalco et al., 2016).

Tasks will also differ in the *criterion content* they are targeting, for example, whether they are asking for the color, shape, or lexicality of a stimulus (Kahneman, 1968). Criterion content can be characterized as the *set of cues* individual observers factually use to perform a task. For instance, when trying to discriminate a masked prime, they may use cues from visual motion, flicker, perceived response conflict, or task knowledge in addition to their actual awareness of the critical feature (*Cue Set Theory*, T. Schmidt & Biafora, 2024). Any two measures that are appreciably different in their criterion contents can be expected to have different decision axes, even though these can be arbitrarily close to each other (e.g., highly correlated) in multivariate space. For instance, studies that compared discrimination and PAS ratings have sometimes found a great deal of convergence between the measures (Kiefer, Frühauf et al., 2023a, 2023b), but at other times striking discrepancies, especially when looking at the behavior of single observers (Biafora & T. Schmidt, 2022). Similarly, two measures as similar as visibility judgments and decision confidence do not necessarily lead to identical results (Rausch & Zehetleitner, 2016; Rausch et al., 2015).

Therefore, when measuring the visibility of a stimulus, we must account for the fact that visual awareness consists of a multitude of facets that must be measured separately and may even undergo double dissociations among each other (see Koster et al., 2020, for some striking evidence). Multiple methods exist to measure even a single facet of visual awareness, either subjectively and objectively, each with its own decision space and criterion content. In visual masking alone, commonplace methods contain stimulus rating, stimulus matching, direct scaling, multidimensional scaling, and, of course, the whole variety of detection and discrimination tasks (Breitmeyer, 1984; Breitmeyer & Oğmen, 2006; Hautus et al., 2022; Sackur, 2013). Different tasks have also been used to address specific criterion contents, asking for position, shape, motion direction, lexicality, object class, and countless other stimulus properties. Consequently, dissociations among direct tasks are abundant in the literature on visual awareness, are of theoretical interest, and warrant extensive study. For instance, metacontrast masking largely spares the ability to detect a target but strongly affects brightness matching and contour discrimination (Breitmeyer & Oğmen, 2006), and targets that are easy to detect can be very difficult to discriminate (Vorberg et al., 2003). Dissociations between indirect tasks, on the other hand, are just as common – for example, responses made with different effector systems, different numbers of response alternatives, or different speed-accuracy instructions.

We therefore agree with Stockart et al. that different types of direct measures should be considered (e.g., objective and subjective ones), that they should be chosen carefully, and that interpretations and inferences should be specific to the measures employed. However, in the light of the extraordinarily rich literature on task dissociations, we find it puzzling that Stockart et al. restrict their recommendations to only two measures, the Perceptual Awareness Scale (*PAS*; Ramsøy & Overgaard, 2004) and forced-choice tasks (obviously, that includes yes-no discrimination [YN] and two-alternative forced choice [2AFC]; Hautus et al., 2022). We have previously criticized the PAS on several grounds (T. Schmidt & Biafora, 2024). Firstly, the original wording of the PAS focuses mainly on detection instead of discrimination of the critical stimulus (and also a bit on decision confidence). It will therefore typically fail to address the specific critical feature that generates the indirect effect. Secondly, when Ramsøy and Overgaard (2004) introduced the PAS, they were careful to apply the ratings to different stimulus dimensions separately (i.e., there were different PAS ratings for color, shape, and position). Without such separation of stimulus aspects, the PAS only focuses on the general visibility of “the stimulus” as a whole, not any particular well-defined facet of awareness. Thirdly, we criticize the common practice of changing the wording of the scale labels from experiment to experiment (expressly encouraged by Sandberg & Overgaard, 2015), which basically obliterates the PAS as a well-defined psychometric tool. Today, “PAS” seems to be an umbrella term for a loose variety of custom-made rating scales that are sometimes not even explicitly described. From a psychometric perspective, any adjustment of the PAS to a novel criterion content creates a new scale with new measurement properties (including its reliability and validity structure).

In contrast, we fully agree with Stockart et al.’s recommendation that interpretations must be specific for the measures employed. Instead of loosely claiming “unconscious perception” or “subliminal priming”, we should state clearly which particular measures are dissociated, and in what way (for instance, “increasing effects of color priming despite chance-level discrimination of prime color”). Importantly, we should be prepared for the discovery that the particular dissociation we observe does not readily translate to other direct or indirect measures (Koster et al., 2020). Because different facets of awareness are interesting in their own right, they should not be viewed as in need of “justification” as “measures of consciousness”. Rather, they should be motivated based on the criterion content they are addressing, for example, detection, color discrimination, lexical decision, and so on. The same applies to the indirect measure.

### Measurement precision and multitasks

*“Use both subjective and objective direct measures.”*

*“Collect all measures on a trial-by-trial basis.”*

*“Ensure adequate precision of the processing and awareness measures.”*

*(from *Stockart et al., 2025*, *Fig. 2*)*

The first two of these lead to a recommendation for dual or even triple tasks. We will argue that they clash with the third recommendation demanding adequate measurement precision.

First of all, we naturally agree with the recommendation that all measurements should be high-powered and precise. We are strongly opposed to the common practice of employing weak, short, and underpowered tests for the direct task. Examples would include restriction to only a short “post-test”^3^, the use of weak criteria for unawareness (e.g., nonsignificant correlations between direct and indirect measures calculated across a small number of participants), or linear extrapolation from clearly discriminable to purportedly unseen stimuli (see Stein et al., 2024, for a forceful critique of such practices). In our own research, our goal is to have enough trials per data point and participant to evaluate direct and indirect effects in single observers, not just as group averages (F. Schmidt et al., 2011). This is why we have a strong preference for designs with a relatively small array of observers but high measurement precision within observers (“small-*N*/large *N*_*i*_” designs”; Smith & Little, 2018; see Arend & Schäfer, 2019; Baker et al., 2021, for showing that such designs can have excellent statistical power even at the group level). The level of individual participants is especially important when visual masking is involved because masking effects vary widely and qualitatively across observers, to a degree that averaging them can be downright misleading (Albrecht et al., 2010; Albrecht & Mattler, 2010, 2012, 2016; Biafora & T. Schmidt, 2022). Many studies in visual masking therefore take care to show the results of individual participants, in line with the research tradition of psychophysics. Of course, we do acknowledge that there are some paradigms and methods that require larger numbers of observers, especially when based on correlations between measures (Vadillo et al., 2024).

As already stated, we concur with the recommendation that subjective as well as objective measures should be used in studying unconscious cognition – but potentially all of them, not only PAS and forced-choice discrimination. For example, Koster et al. (2020) first asked participants to describe their subjective percepts of metacontrast-masked shape primes. Next, the reported percepts were sorted and classified, and the resulting ratings were used as direct tasks in a second part of the study. It turned out that different percepts had qualitatively different time courses. For instance, some increased with prime-mask SOA, some decreased, and some increased only when prime and target were consistent in shape but not when they were inconsistent. Objective discrimination performance always increased with SOA and was therefore double-dissociated from several of the other awareness measures. This seminal study clearly demonstrates that different measures can be based on widely different criterion contents and that no single measure will be able to capture the contents of subjective awareness as a whole (T. Schmidt & Biafora, 2024). This is true for both measures advocated by Stockart et al., forced-choice discrimination and PAS ratings.

The second recommendation, however, seems problematic. Collecting direct and indirect measures “on a trial-by-trial basis” is supposed to mean “on the same trial”, i.e., in a dual or even triple task. For instance, Peremen and Lamy (2014) asked participants to perform three tasks on each trial of several masked-priming experiments: first, a speeded response to the pointing direction of the target (2YN), then an unspeeded response to the direction of the prime (2YN), and finally a PAS rating (on an apparently modified scale not fully described). Biafora and T. Schmidt (2022, Exp. 1) compared such a triple task to the corresponding single tasks. The experiment had six sessions. The first three were entirely devoted to single-task measurements of the target response, the prime response, and the PAS rating (in its original wording), respectively. In the final three sessions, all three tasks were performed concurrently on each trial in the same sequence as in Peremen and Lamy (2014). We found that the triple task completely changed the pattern of results in all three measures involved. Compared to single tasks, the triple task yielded much higher prime discrimination performance, but without a correspondent increase in PAS ratings. More importantly, it drastically altered the temporal structure of the priming effects. The triple task led to a striking 160-ms delay in response time to the target, a marked reduction in fast errors, and a reduction in the time-locking of errors to the prime. Note that these changes all indicate that response priming lost its usual characteristics of fast sequential feedforward processing of primes and targets. Even more importantly, there were qualitative changes in data patterns. While both single and triple tasks showed decreasing (type B) masking functions in both direct measures, priming effects increased in the single task but *decreased* in the triple task – a data pattern extremely unusual in response priming. As a result, a highly informative double dissociation between priming and masking (stronger priming in spite of decreasing discrimination) turned into an uninformative positive association because priming effects suddenly developed in an implausible direction. Kiefer, Harpaintner et al. (2023a, 2023b) and Wentura et al. (2025) report similar problems in the domain of semantic and affective priming. We concluded that the multiple responses per trial had to be collected and maintained in working memory before being reproduced in sequence, and that this operation simply alters (and damages) the nature of processing for all variables involved. We analyzed the data at the group level, but also observer by observer, and found many surprising dissociations between forced-choice performance and PAS ratings: Whereas prime discrimination performance was generally higher in the multitask, PAS ratings were higher in some observers but lower in others.

The higher discrimination accuracy in the triple compared to the single task may have been a result of differential practice (the triple task was always performed in the later sessions because we expected it to require sufficient practice in the single tasks). However, there is always a danger that reconciling the information from all the concurrent tasks may provide additional cues to the identity of the prime. For example, if participants developed the (correct) hypothesis that inconsistent primes provoke response errors, one successful strategy for improving prime discrimination in multitasks would be to choose the response opposite to the target on each error trial. Relatedly, Li et al. (2024) have shown that concurrent confidence ratings can reactively improve accuracy but prolong response times. They explain this by more conservative boundary settings in a drift-diffusion model.

The conclusion from our multitask studies was that triple tasks (and possibly even dual tasks) are not well suited for measuring the relations between priming and masking because they impose a massive cognitive load that impairs all measurements (Biafora & T. Schmidt, 2022; see also Kiefer & Kammer, 2024). It is, however, theoretically interesting to see *how* and *why* those measurements are impaired. This question is especially interesting in the light of an argument by Peremen and Lamy (2014), who attempt to turn our own reasoning upside down. They suggest that the double dissociations observed between increasing response priming and decreasing mask discrimination (Vorberg et al., 2003; Yang & Rouder, 2025) are actually an artifact of attending to the target in one task but to the prime in the other. Such attention allocation has traditionally been regarded as a good thing because it ensures that both priming and prime discrimination are measured under optimal attentional conditions, yielding a conservative test of perception without awareness. However, in Peremen and Lamy’s view, only multitask measurements allow for an unbiased comparison between priming and masking because they somehow equalize those attentional differences. Apart from the fact that this argument calls the entire literature on visual masking and response conflict paradigms into question, which usually employs single tasks, it is unclear why the equalization of attention should work in the first place. It seems more likely that attention is selectively withdrawn from the target because the prime-related tasks are more cognitively demanding. In any event, the massive time delay introduced by the multitask situation presents a problem for both response priming and masking. In priming, slower responses are associated with response inhibition (*negative compatibility effect*; Eimer & Schlaghecken, 1998) as shown by analyses of response time distributions (Panis & T. Schmidt, 2016; T. Schmidt et al., 2022) and pointing movements (T. Schmidt et al., 2015). And in metacontrast masking, masking functions can change from increasing (*type A*) to U-shaped (*type B*) when observers are required to respond more slowly (Lachter & Durgin, 1999). These findings suggest, unsurprisingly, that it is rather the triple task that tends to invite artifacts. For these reasons, the recommendation for multitasks not only clashes with the requirement of “adequate precision”, but also with basic measurement validity.

No matter whether one prefers single tasks or multitasks – direct and indirect tasks should be explored in their own right and according to their specific characteristics and requirements. This means that researchers interested in unconscious processing need to pay attention to quality standards in adjacent fields like visual masking, visual rivalry, semantic priming, or the continuum of response priming, flanker, and inhibition effects (Ansorge et al., 2014; Breitmeyer & Öğmen, 2006; Ellinghaus et al., 2024; Panis et al., 2016, 2020; Sterzer et al., 2014). For instance, many experiments have analyzed the time course of response activation and inhibition in response priming (e.g., Lingnau & Vorberg, 2005; F. Schmidt & T. Schmidt, 2010; T. Schmidt et al., 2022; T. Schmidt & F. Schmidt, 2009; Vorberg et al., 2003; Wolkersdorfer et al., 2020). All of these experiments were carried out as single tasks, and many of them employed unmasked primes to explore the properties of response priming without the additional complication of masking effects (e.g., Haberkamp et al., 2013; F. Schmidt & T. Schmidt, 2014; Seydell-Greenwald & T. Schmidt, 2012). These studies suggest that response priming is based on strictly sequential waves of response activation elicited in turn by prime and target, thus forming a very simple system of sequential feedforward activation. Understanding the feedforward nature of unmasked response priming then helped explain the dissociation between priming and masking. Research on backward masking had already led to evidence that it is a time-delayed process based on recurrent or reentrant signals (Breitmeyer & Öğmen, 2006; Breitmeyer et al., 2004; Di Lollo et al., 2000; Fahrenfort et al., 2007; Francis, 2000; Kammer, 2007; Lamme et al., 2002; Pascual-Leone & Walsh, 2001; see also Becker & Mattler, 2019). Hence, it was plausible that an initial, prime-triggered feedforward wave of motor activation might simply escape the slower masking process (*Rapid-Chase Theory*, T. Schmidt et al., 2006). From this perspective, the massive time delay and loss of time locking under multitask conditions is, of course, alarming: Even if “priming effects” continue to be found (Peremen & Lamy, 2014), they are not necessarily comparable to the response priming effects usually studied. This example of theoretical integration across fields illustrates that in order to understand masked priming, we not only have to understand masking, but priming as well. To us, that implies that the methods we employ in research on perception without awareness should not be too disparate from those employed in adjacent fields. For instance, if we employ all measures of priming and masking in dual or even triple tasks, we deviate strongly from the standards under which these variables are usually measured in specialized fields and can no longer rely on the knowledge that has been accumulated about those tasks.

### The post hoc selection fallacy

*“Collect all measures on a trial-by-trial basis.”*

*“Examine the possible effect of misclassification due to measurement error.”*

*(from *Stockart et al., 2025*, *Fig. 2*)*

It may not be apparent at first glance that these recommendations deal with the strongly criticized practice of post hoc selection of supposedly “unconscious” trials on the basis of visibility ratings. Taken singly, both recommendations reached a strong consensus in the group of coauthors. There was, however, no explicit vote on the practice of post hoc selection itself.

Stockart et al. extensively discuss the well-known problems of regression to the mean (RttM) arising in post hoc selection (Shanks, 2017; see also Malejka et al., 2021; Rothkirch & Hesselmann, 2017; Rothkirch et al., 2022; Shanks et al., 2021; Vadillo et al., 2022). When trials from the lowest rating category are selected, unreliability of the scale causes some of those trials to land in the lowest category only because of noise. As a result, the visibility of those trials is underestimated (conversely, the visibility in the highest rating category is overestimated). This phenomenon is sometimes called a *squeeze effect*: no matter whether scores are extremely high or extremely low, they tend to be more moderate upon replication. Characteristically, squeeze effects also occur retrospectively: extreme scores in the replication turn out to have been more moderate in the first run. Regression to the mean is an inevitable consequence of any less-than-perfect reliability in the direct measure, and the lower the reliability, the stronger the squeeze (Campbell & Kenny, 1999).

Stockart et al. propose some mathematical correction techniques for regression to the mean. However, they never discuss the consequences of the simple dissociation paradigm they advocate. First of all, the techniques proposed so far often require relatively strong assumptions to achieve the correction. For instance, Dienes (2024) assumes that there are only two perceptual states (conscious or unconscious), that there is no response bias in their subjective classification, and that misclassification rates follow a particular Bayesian prior function (but see Yaron et al., 2024, for a nonparametric approach). Second and more important, all those corrections move the estimate away from the point of zero awareness. Corrections for regression to the mean therefore make it even harder to find a simple dissociation: Not only do we have to be lucky in producing an initial estimate that falls as closely as possible to the zero-visibility line, but it also has to remain sufficiently close after correction for RttM. Stockart et al. appear quite optimistic about the possibility of correcting for the regression artifact, but fail to mention that the correction will likely destroy the simple dissociation.^4^

However, regression to the mean, as annoying as it is, is not the greatest problem here. In an earlier critique of post hoc sorting, the practice was characterized as a “sampling fallacy” and as “capitalizing on chance” (T. Schmidt, 2015). Stockart et al.’s suggestion of collecting both a forced-choice response and a PAS rating on the same trial allows for a striking illustration of this fallacy if we realize how those two measures are psychophysically related. Consider the familiar signal-detection model of a simple yes-no discrimination task where one of two stimuli is presented on each trial and the participant has to decide under uncertainty which one it is. The standard SDT model postulates that the internal evidence for one versus the other stimulus alternative is measured by two overlapping distributions, S1 and S2 (Fig. 2a, left panel). Sensitivity (*d’*) is defined as the standardized distance between the distributions (i.e., by their degree of overlap). Variation within the distributions is assumed to arise solely from random noise. The two distributions vary along the horizontal *decision axis* that ranges from evidence in favor of S1 to evidence in favor of S2, with a point of indifference in between. For the objective discrimination task, a criterion is placed on the decision axis, and responses for S1 or S2 are chosen depending on which side of the criterion the evidence falls on any given trial. For a signal-detection analysis, we can arbitrarily define *hits* when Stimulus 2 is correctly identified as “Stimulus 2”, and *false alarms* when Stimulus 1 is incorrectly identified as “Stimulus 2”.

There is a standard way for representing visibility ratings within this decision space. Stockart et al. correctly recommend that both the PAS labels and the forced-choice task should be designed to reflect the critical feature that generates the indirect effect (as originally intended by Ramsøy & Overgaard, 2004). This helps to bring the decision axes for PAS ratings and forced-choice discrimination into register so that both tasks ideally use the same axis. This leads to the SDT standard model for rating experiments, where the different rating categories will cut the plot into vertical stripes (Fig. 2a; Gescheider, 1997; Green & Swets, 1966; Hautus et al., 2022). The lowest ratings are placed in the center of the plot where the overlap between the distributions is largest; the next highest ratings come from the two stripes left and right to the center category; and so on towards the tails of the distributions where visibility ratings are highest. The different PAS categories are separated by their own criteria that determine in which category a rating is placed. Those categories can be used to plot the cumulative hit rate against the cumulative false alarm rate in a receiver operating characteristic (ROC) curve. The curve starts in the lower left corner with the high-visibility ratings for Stimulus 2, continues through progressively lower ratings for Stimulus 2, switches to low ratings for Stimulus 1, and ends on the upper right with the high-visibility ratings for Stimulus 1 (Fig. 2b, left panel). Note that if the standard SDT model applies (normal distributions, equal variances), we do not even need low visibility ratings from any actual observer: From our knowledge of *d’* alone the entire ROC is determined, including the point where it crosses the minor diagonal of the plot (marked with a “+” in Fig. 2b). This is the theoretical point of indifference where an optimal observer (here, one with a symmetrical response criterion) would have no idea whether S1 or S2 was presented.

Figure 2a illustrates an idealized case in which both the discrimination task and the PAS ratings explicitly address the same critical feature (e.g., the prime’s shape). In most experiments, the critical feature is uniquely determined as the feature that defines the indirect effect (T. Schmidt & Biafora, 2024). For example, consider an indirect task asking to indicate as quickly as possible whether the target is a square or a diamond. Then the corresponding direct discrimination task would ask participants to indicate whether the prime was a square (S1) or a diamond (S2), and if participants follow those instructions, their decision axis should be as depicted in Fig. 2a. To align the PAS ratings with that decision axis, we have to take care that the ratings also address the critical feature (square vs. diamond). The scale labels should thus range from *“1: no idea whether it was a square or diamond”* to *“4: absolutely certain which one of the two shapes it was”*. The PAS scale in its original wording, which only refers generically to “the stimulus” or even just “something”, does not ensure that the two measures have comparable contents. If they do not, the decision axes for the two direct measures will be misaligned, the PAS ratings cannot be used for constructing the ROC, and the interpretational problems will be greatly aggravated.

If the direct task is detection instead of discrimination, we compare a noise distribution N (on the left of the decision axis) and a signal-plus-noise distribution S (to the right), with their normalized distance *d’* defining the observer’s detection sensitivity (Fig. 2a, right panel). Now hits and false alarms are defined as correct responses to S and incorrect responses to N, respectively. PAS detectability ratings will cut this detection space into four vertical stripes, from PAS = 1 on the far left to PAS = 4 on the far right. If the standard model is correct, the overall sensitivity determines every data point to lie on the theoretical detection ROC, the low-visibility and the high-visibility ratings alike (Fig. 2b, right panel). Note that response bias plays a larger role in detection than in discrimination, and the placement of the rating criteria along the decision axis is at the observer’s discretion. Also note that we once again assume that the decision axes for detection judgments and PAS detectability ratings are in register by forcing the rating categories to explicitly address the prime’s detectability, not its discriminability. T. Schmidt and Biafora (2024) warn against using detection as a direct task. For instance, detection tasks usually do not have a comparable counterpart in the indirect task, and they may be based on stimulus cues other than the critical feature.

Strikingly, in either of these standard models the PAS ratings do *not* represent different levels of perceptual sensitivity for the prime. Sensitivity (*d’*) is defined at the level of the entire *distribution* of trials in an experimental condition; once known, it completely determines the shape of the ROC, and the individual PAS categories merely correspond to different response criteria along that single ROC. This is why there can always be many response criteria, but only one underlying sensitivity per condition. In contrast, the selection of individual PAS ratings is carried out on the basis of *single* trials. Thus, selecting trials from one PAS bin rather than another merely isolates differences in response bias, irrespective of the underlying sensitivity.

For a discrimination task, the lowest PAS ratings come into play near the center of the ROC where the curve crosses the minor diagonal (the theoretical point where response bias is zero). This means that low PAS ratings from individual trials are not indicative of low sensitivity to the stimulus; they can, in fact, only be interpreted if the underlying condition-wide sensitivity to the stimulus is known. In addition to measuring visibility on every single trial, it is therefore necessary to consider the *overall* sensitivity from the entire *distribution* of trials in any experimental condition.

**Fig. 3:**
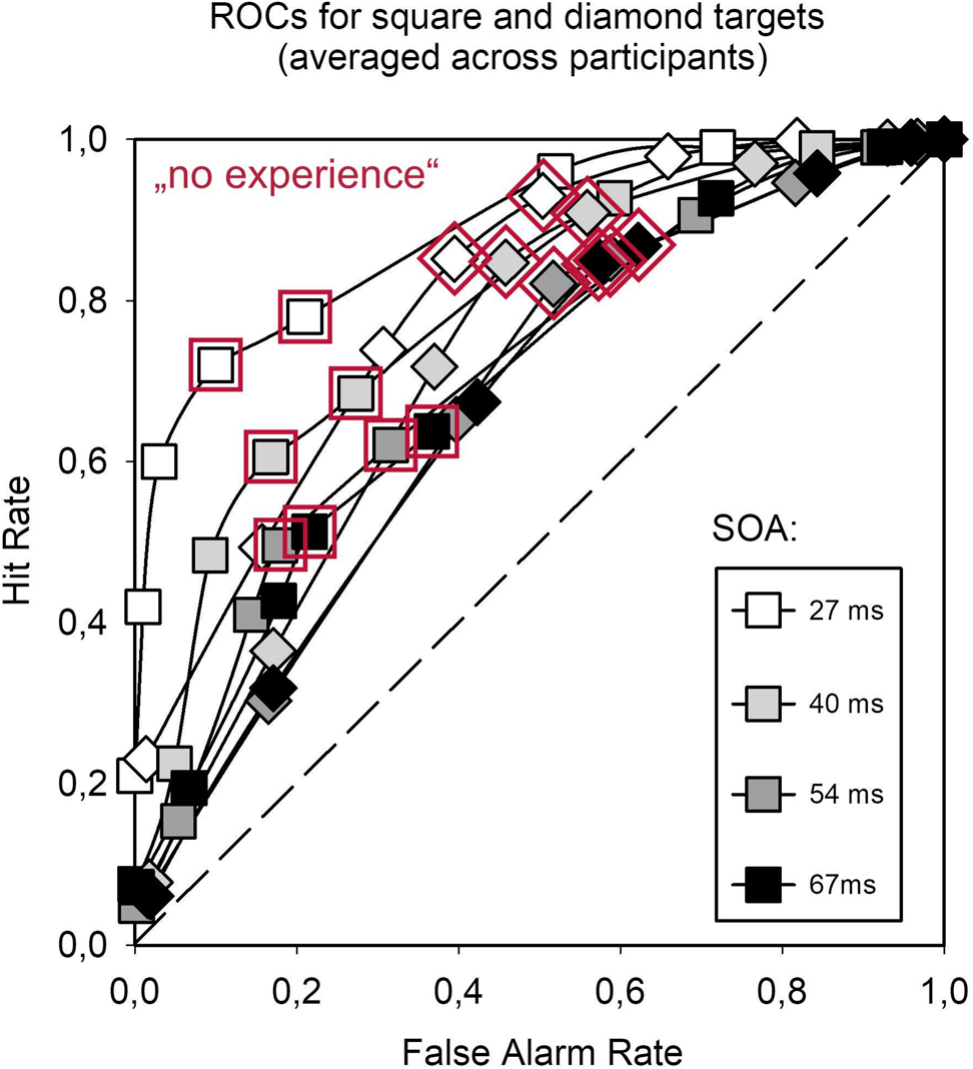
Receiver operating characteristics from Biafora and T. Schmidt (2022, Exp. 1, triple-task condition). ROCs are constructed separately for the different prime-target SOAs (*shading*). The decrease in sensitivity with SOA illustrates type B masking. ROCs are plotted separately for the two different target shapes (*squares* or *diamonds*), because averaging across target identities would lead to underestimation of sensitivity due to response bias (Vorberg et al., 2004). ROC points indicating ratings of “no experience” (PAS = 1) are highlighted in *red*. Note that there are two of them on every curve, corresponding to the two central rating categories (–1/+1) in Fig. 2

This analysis makes clear why the isolated analysis of low PAS ratings is a sampling fallacy: it constitutes a negative selection from an underlying distribution that could be consistent with different levels of sensitivity – and that may or may not be consistent with the hypothesis of unconscious processing. In our own data (Biafora & T. Schmidt, 2022, Exp. 1), PAS ratings of 1, 2, 3, and 4 corresponded to prime discrimination accuracy *p*_*c*_ of.590,.643,.722, and.933, respectively (averaged across all observers and conditions). Post hoc sorting by PAS ratings would therefore conclude that discrimination accuracy in the lowest rating category was at 59.0 % (which would correspond to *d’* ≈.455, using the approximation that *d’* ≈ *z*(*p*_*c*_) – *z*(1-*p*_*c*_); Hautus et al., 2022). Even if correct, this value would be too high to meaningfully speak of unconscious stimulus conditions, but it is in fact a gross underestimation of the true underlying accuracy across all ratings, which is 68.8 % (*d’* ≈.980). Figure 3 shows the corresponding ROCs separately for the four SOAs and for both types of target shapes. This simple example makes clear that low PAS ratings cannot be interpreted in isolation (T. Schmidt, 2015). They may successfully isolate trials with low subjective *visibility*, but those can be expected to occur at *any* level of sensitivity – after all, every discrimination ROC has to traverse a point of indifference about the identity of the prime! Therefore, low PAS ratings do not isolate trials with low *sensitivity*, which remains unknown unless the entire distribution of trials is considered. They only isolate trials with neutral *response bias*, a property orthogonal to sensitivity.^5^

**Fig. 4:**
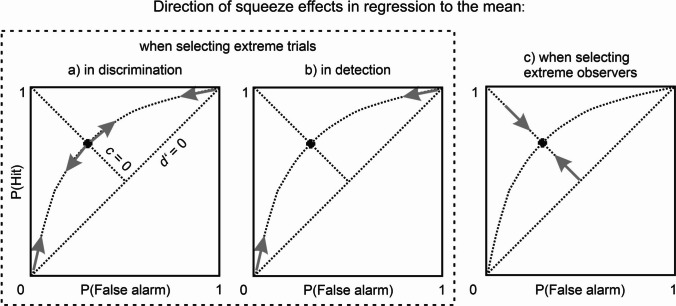
Direction of squeeze effects when regression to the mean occurs on the basis of selective analysis. **a)** When selectively analyzing trials with very low or very high visibility ratings in a discrimination task, squeeze effects are directed away from extreme ratings (like ±1 or ±4) and towards the center of the trial distribution for each of the two prime alternatives. **b)** In a detection task, only squeeze effects from the extreme ends towards the center would be expected because the medium categories are usually not candidates for selection. **c)** When selectively analyzing observers with very low or very high performance, squeeze effects are directed away from extreme performances and towards the true underlying sensitivity averaged across observers. This would be expected for both discrimination and detection

Regression to the mean contributes to this problem, but probably not in the way users of post hoc selection would expect. The artifact occurs because an imperfect reliability in the PAS ratings causes some single trials to be misplaced into extreme rating categories. Upon retesting, the regression artifact would lead them to be squeezed back into categories closer to the center of the trial distribution (Fig. 4a, b). Importantly, regression to the mean in the PAS ratings does not change the underlying ROC, it only leads to misclassification *along* the curve: it affects only trial-by-trial visibility ratings, but not condition-wide sensitivity. It is important to contrast this effect with the other widespread practice of discarding entire participants on the basis of their objective discrimination performance. Selectively analyzing low-performing observers constitutes a negative selection of observers, and once again unreliability in the measurement leads to regression to the mean. However, this time, it works in the direction of sensitivity, not visibility (Fig. 4c): by selecting only low-performing observers, we underestimate the performance they would show upon retesting.

What is true for discrimination tasks (2YN and 2AFC) also holds for detection and “liminal prime” tasks. When selecting detection trials with extreme ratings, squeeze effects are directed from the ends of the curve towards the center (in detection, the central rating categories would normally not be regarded as interesting candidates for selection). In contrast, when selecting *observers* with extreme performance, squeeze effects are directed towards the true underlying sensitivity just as in the discrimination task. – In the liminal-prime paradigm (Avneon & Lamy, 2018; Bachmann & Francis, 2014; van den Bussche et al., 2013), the same weak stimulus is presented over and over again, and the variance in visibility ratings is assumed to arise from spontaneous fluctuations in visibility. Because of the lack of a comparison condition (e.g., an S2 or N), this task design is principally unable to separate sensitivity and response criteria. In such a study, which is no longer experimental but merely correlational, the outcome may be determined exclusively by measurement noise.^6^

Proponents of subjective criteria of awareness may complain that we insist on an “objective” sensitivity standard as a precondition for subjective ratings, but that would be a misunderstanding. SDT is agnostic about any concepts of consciousness or subjectivity, and the decision axis in Fig. 2a may well be conceived as genuine subjective information for S1 versus S2 or for signal versus noise. The critical question is not whether an objective measure is a requirement for the interpretation of a subjective one, but whether there is any clash between a selected subset of measurements *on the level of single trials* and the overall performance *on the level of the distribution*. Objective sensitivity as measured by *d’* is only one example of a distribution-wide measure, but any other distribution-wide measure could create the very same problem, even a subjective measure. For instance, consider an experiment where we present each stimulus condition in a homogenic block of its own. In addition to measuring PAS ratings and response times on each trial, we ask observers to rate the average visibility of the entire block on the PAS scale. Then we could have the same discrepancy between trial-wise selection and condition-wise measurements when a selection of PAS = 1 trials clashes with a blockwise PAS rating of, say, 2 or 3.

### The stripe-by-stripe fallacy

We have seen that in discrimination as well as detection tasks, trial-wise measurements at different response criteria (like the lowest PAS rating) can only be interpreted if the condition-wide sensitivity is known. However, many studies have attempted to measure sensitivity separately for different rating categories without realizing that sensitivity is a property of the *distribution* of trials, not of single trials. Many computational artifacts can arise from calculating indices of sensitivity separately for different rating categories. Again, consider Fig. 2a (left panel), where the rating categories are numbered from negative to positive, and let *A*_*i*_ equal the percentage of correct responses in rating category *i*. When *d’* = 0, the S1 and S2 distributions are identical, and *A*_*i*_ equals 0.5 in each rating category (chance level guessing). However, whenever *d’* is not zero, the lowest rating categories will always have lower accuracy than the highest rating categories, just like we have seen above in the data from Biafora and T. Schmidt (2022). This occurs by necessity because the lowest rating categories will come from the area of largest overlap between the S1 and S2 distributions, while the highest rating categories come from the tails where errors are rare. However, it would be a mistake to interpret any of those seemingly different accuracy levels in isolation: none of them is free to vary because they are all jointly determined by the degree of overlap between S1 and S2 (i.e., by sensitivity). By the same argument, Stein et al. (2016; see also Fahrenfort et al., 2024) have shown that it is a mistake to calculate *d’* (or any other sensitivity statistic) separately for each rating category, even though this is frequently seen in the literature (e.g., Peremen & Lamy, 2014; Soto et al., 2011; Wixted et al., 2015; see Soto & Silvanto, 2016, for a reply to Stein et al. that failed to convince us). Those statistics will be systematically too small for the rating categories in the center of overlap and systematically too large for the rating categories in the periphery. Similar distortions would occur in a detection task (Fig. 2, right panels).

**Fig. 5:**
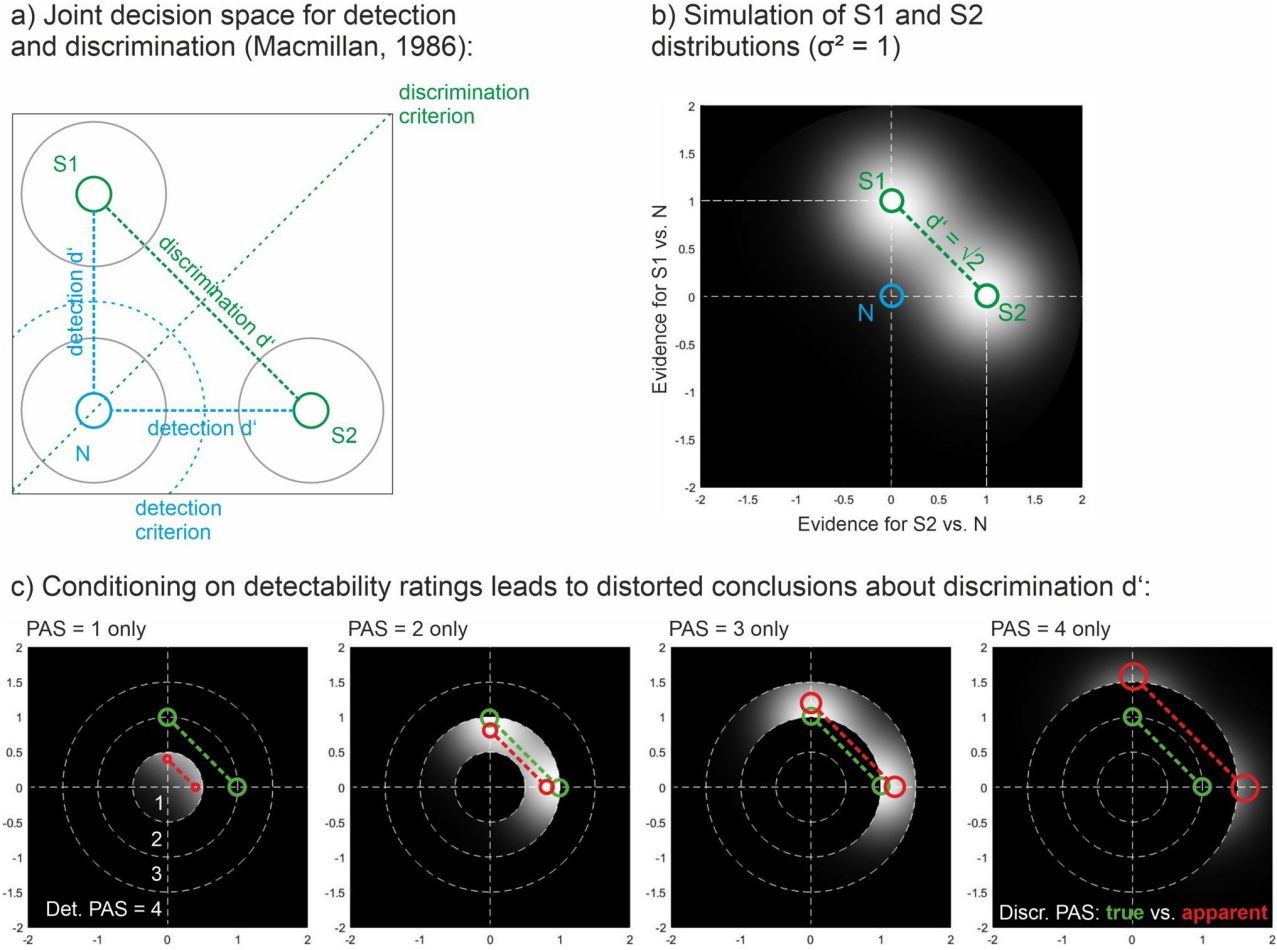
**a**) A joint decision space for discrimination and detection tasks (Macmillan, 1986). For discrimination (*green*), the criterion is chosen to separate the S1 and S2 distributions. For detection (*blue*), the criterion is chosen to separate both S1 and S2 from noise (N). Note that the two criteria are orthogonal. **b)** S1 and S2 simulated as bivariate normal distributions with unit variance. Distributions are relative to the center of the noise distribution (N). Because the distance between N and S distributions is one standard deviation unit, the true distance between S1 and S2 is √2. **c)** Detectability ratings (PAS from 1 to 4) can be represented as concentric regions around the N distribution. Selective analysis of detectability ratings truncates the underlying S1 and S2 distributions and distorts their apparent distance and degree of overlap. For low detectability ratings, discrimination *d’* appears too low; for high detectability ratings, it appears too high. *Green circles*: true means of S1 and S2, always √2 units apart. *Red circles*: apparent peaks of the truncated distributions after conditioning on detectability ratings

So far, we have only considered the case where discrimination responses and visibility ratings share the same decision axis, which, in case of the PAS, requires that participants are explicitly instructed to judge the critical feature that is the target of the discrimination task. Is the stripe-by-stripe fallacy restricted to this case, or does it also occur when visibility ratings and discrimination judgments are orthogonal? An interesting case in point is the use of *detectability ratings* in conjunction with discrimination responses, especially because the PAS, in its original wording, is mostly a detectability scale (T. Schmidt & Biafora, 2024). Macmillan (1986) proposed a joint decision space for discrimination and detection judgments (Fig. 5a). The decision space is two-dimensional and features three distributions: two for the two signals to be discriminated, and one for noise. In discrimination (green), the task is to tell S1 from S2, and the discrimination criterion is chosen to optimally separate those distributions (here, we chose the major diagonal). In detection (blue), however, the task is to tell both S1 and S2 from noise, and any criterion for detection or detectability must be placed somewhere around the N distribution (here, we chose concentric circles). Without loss of generality, we only consider the simple case (Fig. 5b) where the major axes of the space are orthogonal, where S1 and S2 are bivariate normal distributions with unit variance, and where the means of S1 and S2 are placed one unit away from the mean of N so that the true distance between S1 and S2 is known to be √2 (green markers in Fig. 5b, c). Figure 5c shows what happens when different detectability ratings are considered in isolation (cf. King & Dehaene, 2014). When only PAS ratings of 1 are considered (“no impression of the stimulus”), we restrict attention to a tight circle around N and “see” only small truncated parts of S1 and S2. Those parts will seem to overlap strongly, and it will be difficult to tell their peaks apart. In the other extreme, when only PAS ratings of 4 are considered, the truncated S1 and S2 distributions will seem to be well separated, with their peaks far apart. Now assume that we calculate discrimination *d’* separately for the different detectability ratings, using only the observations within each concentric circle. Then it is clear that we will *underestimate* discrimination when detectability ratings are low, but *overestimate* it when detectability ratings are high (red markers in Fig. 5c). Even though other geometries of the decision space are possible (correlated distributions, unequal variances, differently shaped criteria, etc.), the problem is fairly general. We conclude that conditioning on PAS ratings *generally* distorts the measurement of discriminability, no matter whether the rating axis is collinear with the discrimination axis or orthogonal to it. Both the post hoc selection fallacy and the stripe-by-stripe fallacy are obviously universal to detection spaces and will occur no matter how participants apply the visibility ratings.

Generally, a simple detection or discrimination experiment can only have one underlying sensitivity because there is only one normalized distance between two distributions of internal evidence. Therefore, all the seemingly different values identified by the stripe-by-stripe method can only be a function of response criterion (bias). This principle applies to all types of performance measures, like percentage of correct responses, various sensitivity statistics like *d’* or *H-minus-F*, and psychometric functions. For example, King and Dehaene (2014), after providing a detailed and correct analysis of the stripe-by-stripe problem, nevertheless report an experiment where participants had to decide between two numerals, with various degrees of intermediate morphs along the decision axis. This experiment would normally yield a single psychometric function for each observer. Yet they report *several* such functions, one for each visibility rating, and conclude that high-visibility stimuli are characterized by steeper psychometric functions than low-visibility stimuli. In fact, the different slopes are a result of the stripe-by-stripe fallacy: By conditioning on the visibility rating, we select subsets at different response criteria, but all from the same underlying sensitivity function. As before, there is only one underlying psychometric function, and the apparent split into several only reflects the different response criteria.

### General discussion: From selective analysis to functional dissociations

The problem of unconscious cognition is a true classic of experimental psychology. Even though the field possesses an unbroken research tradition dating back into the late 19th century, it was marred by boom-and-bust cycles in which a resurgence of interest in the topic first led to an attrition of methodological criteria and then was ended by seminal review papers that called the validity of the entire field into question (e.g., Eriksen, 1960; Holender, 1986). Both times this happened, the field collapsed and fell into disregard, if not disrepute, for a decade or more. If there is any lesson from the history of our field, it is this: Convincing each other that our methods are “recommended” or “good practice” is just not good enough. The ambition must be to convince hardcore skeptics (past, present, and future) that our methods are valid and can stand the test of time. This is only possible by using strict standards of measurement, experimental design, and psychophysical modeling, and by avoiding well-known statistical artifacts that would bias our conclusions towards the “wild hypothesis”. If methodological standards in unconscious cognition systematically fall short of what is expected in adjacent fields like masking or response time research, and if we neglect basic principles of statistics, psychophysics, and psychometrics, then our field is increasingly in danger of encapsulation and ceases to be interesting, relevant, or even respectable to others.

Stockart et al.’s attempt to reach a consensus among researchers from various schools and methodological preferences leads to many recommendations that we share. However, in its current form, the project condones and even advocates the widespread practice of post hoc selection on the basis of multiple responses per trial. However, as we have shown here, post hoc selection is not a valid technique for isolating trials without awareness. At the heart of the problem is a sampling fallacy: selection on the basis of a *sample* does not change the properties of the *population* on which the sample is based (T. Schmidt, 2015). Even though subjective visibility may genuinely fluctuate from trial to trial, and even if a rating scale validly captures those fluctuations, an observer’s sensitivity must still be calculated *across* trials and may easily be too high to plausibly argue for unconscious cognition. If sensitivity is represented by the shape of the ROC curve, then the lowest visibility ratings simply represent the points on the curve that are associated with the largest subjective uncertainty. Still, those points are part of the very same ROC as all the higher visibility ratings. In discrimination tasks, they lie right in the center of the curve and could be obtained by interpolation even if low PAS ratings never factually occurred – after all, *any* ROC has to pass the minor diagonal.

Relatedly, selecting any particular visibility category and calculating separate sensitivity statistics for that subsample is a miscalculation of psychophysical measures. It results in values that are too low when subjective visibility is low and too high when subjective visibility is high (the stripe-by-stripe fallacy; Stein et al., 2016). Unreliability in the rating scale contributes to these problems by introducing regression to the mean, which leads to an underestimation of visibility at low ratings and an overestimation at high ratings (Shanks, 2017; Shanks et al., 2021). Regression to the mean particularly threatens the validity of low-visibility ratings, which, when isolated, capitalize on the noise in the measurement (T. Schmidt, 2015). However, it is the selection process itself that is invalid. Whenever visibility ratings come from a stimulus condition with an ROC that is too steep, then sensitivity in that condition is likewise too high, even if some of its trials received a low rating. Note that the fundamental problem here is not one of objective versus subjective measures, but one of trial-by-trial versus condition-wide measures: If we used a subjective measure instead of objective *d’* to evaluate an entire stimulus condition, that measure may speak against unconscious processing as well.

All these problems apply not only to discrimination tasks, but also to detection tasks (where rating categories are just arranged differently along the detection ROC) and the “liminal-prime” paradigm (which cannot tell signal from noise in the first place and may be driven entirely by measurement noise). Needless to say, these problems also apply to the selective analysis of binary “seen/unseen” ratings, “seen/guessed” ratings, or confidence ratings. They arise from a fundamental flaw in the research approach: instead of controlling visibility experimentally, one first leaves it to chance and then turns one of the dependent variables (visibility rating) into an independent factor for the analysis of another dependent variable (e.g., response time effect). Using a dependent variable as if it were experimentally controlled is always a flirt with disaster in data analysis. It often leads to unexpected artifacts as the orderly and balanced experimental design is degraded into an unbalanced correlational design, with its usual share of unforeseen conditional probabilities and ill-understood confounds. However, as soon as visibility is under *experimental* control, many of the potential biases and restrictions of visibility measures cease to be so threatening. For instance, Skóra et al. (2021) argue that *absolute* PAS ratings are affected by response bias but that *relative* ratings are not: PAS profiles may change in absolute magnitude but can still be compared across conditions. Such data indicate that there is a useful function for PAS-like rating scales as long as the entire rating profile is compared across experimental conditions. In contrast, if we wanted to use those ratings as a basis for post hoc selection, any such shift in absolute magnitude would destroy the validity of the selection.

Functional dissociations between direct and indirect measures solve most if not all of those problems. First, there are many dissociation patterns that neither require nor benefit from null sensitivity in the direct measure (they are literally “beyond the zero-awareness criterion”, T. Schmidt, 2007). Second, they all utilize parametric variations of stimulus parameters like stimulus intensity or prime-mask SOA, providing research designs with internal validity and ensuring that functions are orderly, well behaved, and measured with precision. Third, they all trace out meaningful functional relationships that are as relevant within our field as they are in more specialized areas (e.g., priming and masking functions).

The most basic type of functional dissociation is an invariance dissociation (or its special case, the simple dissociation), where one of the measures is shown to remain invariant (or close to zero) as the other one is experimentally varied. For instance, response priming increases with prime-target SOA even as prime discrimination performance is invariant at chance level (Vorberg et al., 2003, Exp. 1; F. Schmidt & T. Schmidt, 2010). Conversely, simple reaction times to the prime remain invariant as the strength of metacontrast masking is experimentally varied (Fehrer & Raab, 1962). Moreover, the time-course of response priming remains invariant no matter whether the ability to discriminate the prime is low, high, increasing, or decreasing (Vorberg et al., 2003; Yang & Rouder, 2025). Because both priming and masking functions are under full experimental control and no post hoc selection is taking place, neither sampling bias nor regression to the mean plays any role. Invariance and simple dissociations are, however, dependent upon the assumption that the invariant measure is exhaustively valid and reliable with respect to the critical feature. Otherwise, the invariance could simply arise from limited sensitivity to the relevant information in the prime. Sensitivity dissociations as demanded by Meyen et al. (2022) are more flexible: they do not require invariance in any of the measures but only that the indirect measure systematically exceeds the direct one (after suitable scaling). Still, however, sensitivity dissociations require an assumption of relative sensitivity regarding the critical feature (T. Schmidt & Vorberg, 2006). Finally, the most informative data patterns come from double dissociations: The demonstration that the same experimental manipulation leads to opposite effects on direct and indirect variables directly shows that they cannot both depend monotonically on the same single source of conscious information. Double dissociation patterns also require the mildest measurement assumptions, because they do not require any assumptions of relative sensitivity, exclusiveness, or exhaustive reliability (see mathematical proofs in T. Schmidt & Vorberg, 2006; see also Biafora & T. Schmidt, 2020; T. Schmidt & Biafora, 2024).

Are there conceptual limits even to double dissociations? Stockart et al. (2025), in response to our commentary, refer to an important paper by Dunn and Kirsner (1988) to point out potential limitations of double dissociations. However, this paper is perfectly in line with our own approach. In essence, Dunn and Kirsner ask whether a double dissociation can still be interpreted as evidence against a single-process model if the polarity of one or both of the measures is unknown. The answer is obviously no, because reversing the polarity of one measure turns dissociation into association, and vice versa. They therefore advocate a data pattern they call “reversed association”: the simultaneous demonstration of an association and a double dissociation between the two measures in different conditions of the experiment. This way, the polarity of neither measure needs to be known, because one of the two patterns will always constitute a double dissociation (even if we do not know which one). Ultimately, the approach simply boils down to demonstrating a double dissociation (the additional association pattern being uninformative) and even uses the same set of assumptions that we have (T. Schmidt & Vorberg, 2006).

Dunn and Kirsner’s approach is part of the more general program of *state-trace analysis* (*STA*; Bamber, 1979), which aims to use dissociation patterns to infer the number of underlying processes (one versus several). Originally, the aim of state-trace analysis was to make such inferences without any assumptions about the monotonicity of the measures involved. However, Ashby and Bamber (2022) conclude that STA is not suited for investigating most dissociation patterns, and that some monotonicity assumptions remain indispensable – with double dissociations as the sole possible exception. Again, the important distinction here is between assumptions of *strict monotonicity* (i.e., exhaustiveness, like the infinitely sensitive barometer) and *weak monotonicity* (like the barometer that may sometimes fail to respond and is only required not to systematically respond in the wrong direction). In our opinion, assumptions of weak monotonicity are benign and indispensable for any type of measurement process, especially if assumptions are made only on the level of expected values; it is only the assumption of strict monotonicity, i.e., exhaustiveness, which is problematic (T. Schmidt & Vorberg, 2006). Whenever the assumption can be made that the direct and indirect measures are at least weakly monotonic functions of awareness, double dissociations immediately refute any single-process model (see proof in T. Schmidt & Vorberg, 2006). This is why Stockart et al.’s (2025) claim that “double dissociations can be accounted for by single process models” (Stockart et al., 2025, section 7) is misleading. Not only does it conceal that an integral assumption required for measurement, weak monotonicity, needs to be violated for such an alternative explanation; it also obscures how peculiar such an explanation would be. Not only would we have to consider an unknown underlying variable explaining both priming and awareness, but one that is contorted in just the right way to trace out the dissociated data pattern point by point across both dependent variables (a data pattern Dunn & Kirsner, 1988, do not even come close to discussing). Ironically, then, Dunn and Kirsner’s arguments against double dissociations apply all the more to simple dissociations with their strong additional assumptions. After all, a simple point dissociation is easy to explain by a single underlying process, especially when compared to a multi-point double-dissociation curve.

A major incentive for using post hoc selection on the basis of multitasks is certainly the desire for a seemingly foolproof method for obtaining trials with unconscious primes, even if only in a fraction of the experiment. In contrast, past researchers working on simple point dissociations were forced to search for a tiny zone of experimental conditions that allowed for just the right amount of priming under just the right amount of masking, often exploiting low measurement precision to argue that discrimination performance for the critical stimulus was not statistically different from chance (Stein et al., 2024). Similarly, researchers employing post hoc sorting and correcting for regression to the mean will have to hope for the indispensable correction to remain close enough to zero to fail statistical significance. We like to call such narrow corridors of measurement conditions “Goldilocks’ hell of statistical indifference”. For a paradigm striving to live in that precarious place, there is always an incentive to accept an underpowered measurement to use the absence of significant direct effects as an argument for unawareness. One advantage of functional dissociations is that they always reward measurement precision because they are usually not confined to a specific value of the direct measure. Additionally, the diverse shapes of dissociation curves between and among various direct and indirect measures are all of theoretical interest – not only highly informative double dissociations, but also well-measured sensitivity and invariance dissociations. Indeed, users of dissociation curves will never have to set foot into Goldilocks’ hell, which is not a good place to be.

From the foregoing, it is evident that post hoc selection must be abandoned because it constitutes a sampling fallacy that capitalizes on chance, generates regression artifacts, and fails to isolate stimulus conditions where perceptual sensitivity is low. Because it actually selects for response bias, not sensitivity, results from that method cannot be interpreted as evidence for unconscious cognition or even as pertinent to the topic. What could a possible future look like for studying visual awareness and unconscious perception? In their study of subjective percepts in metacontrast-masked primes, Koster et al. (2020) have demonstrated a number of functional double dissociations among several different subjective percepts, all of them measures of awareness. Findings such as that make clear that there cannot be a single measure to capture all of “consciousness”: There are many different aspects of visual awareness that should be studied in their own right (Fazekas & Overgaard, 2018; Klein & Hohwy, 2015; Seth et al., 2008). Conscious percepts like perceived brightness, color, or motion require their own specialized theoretical frameworks anyway (Irvine, 2017; T. Schmidt & Biafora, 2024). Functional dissociations offer a research program for seriously comparing different aspects of visual awareness under different cognitive tasks. Solidly grounded in measurement theory, they allow us to unfold the traditional dissociation paradigm by utilizing the entire *D-I* space, not just single points on a single line. Making use of the full range of functional dissociations allows us to conduct successful experiments in a reliable and predictable way – not just by sheer luck, by capitalizing on chance, or by exploiting a statistical artifact, but by the planful study of theoretically meaningful relationships between well-controlled and well-measured variables. Such an approach will always stand the test of time.

## Data Availability

The data used for creating the ROC curves in Fig. 3 can be accessed here: https://osf.io/wm63a/?view_only=6e9277156e7a4d098eaa54728b639f8b

## Footnotes

1 Stockart et al.’s paper first appeared as a preprint (Stockart et al., 2024) that was updated several times. We responded to the first of those versions and then updated our preprint along with Stockart et al.’s updates (T. Schmidt et al., 2025).

2 There are several constructive ways to deal with idiosyncratic masking functions. In metacontrast masking, individual masking functions are stable over time, and observers can often be classified into having increasing (type A) or U-shaped (type B) masking functions (Albrecht et al., 2010; Albrecht & Mattler, 2010, 2012, 2016). So, one avenue would be extensive pretesting under a set of predetermined criteria, followed by contrasting groups of pretested and re-validated observers to demonstrate increasing priming for masking functions of both directions. A second strategy is to take the individual level more seriously: In any group of well-measured observers, there is usually plenty of evidence that the time-course of masking does not predict the time-course of priming. It is straightforward to integrate such evidence statistically across single observers.

3 In the polls on which the recommendations are based, the collective was faced with an improper alternative regarding the question “When should the objective test be administered?”. The alternatives were named “Trial-by-trial”, “During the main task, in separate trials”, and “Post-test”. While this wording is unclear enough, the obvious fourth alternative of spending as much time (or even more) on the direct as on the indirect task was never offered.

4 The technique proposed by Goldstein et al. (2022) is an example of correction in the opposite direction. It first involves the classification of observers as either “conscious” or “unconscious”, and then actually *replaces* the observed accuracy scores of the “unconscious” group by chance performance. Such a procedure biases data analysis towards the desired outcome.

5 Our ROC curves show obvious evidence for response bias: The ROC ratings for “no experience” lie far off the minor diagonal and form distinct clusters depending on the shape of the target. This indicates that the point of indifference (and possibly the shape of the underlying distributions) is biased: Participants do not act like ideal observers but tend to identify the prime with the shape of the visible target. Note that this “contamination” by the target (Stockart et al., 2025) is harmless because response bias can be measured independently of sensitivity. Patterns of response bias can also be quite interesting for understanding participants’ task strategies (Schmidt, 2000). Note that methods for eliminating response bias, like the use of neutral or absent targets, require measuring prime discrimination in separate trials.

6 Another problem of the liminal-prime paradigm is that many sources of spontaneous fluctuations can be expected to affect indirect measures as much as direct measures. For instance, fluctuations in bottom-up signal strength should affect all information processing downstream, and top-down modulations of visual attention have been shown to affect response priming (F. Schmidt, and T. Schmidt, 2010); T. Schmidt & Seydell, 2008).

## Code availability

There is no program code associated with this project.

## Declarations

### Open practices statement

The data used for creating the ROC curves in Fig. 3 can be accessed here: https://osf.io/wm63a/?view_only=6e9277156e7a4d098eaa54728b639f8b

### Author note

We thank Melanie Biafora for the use of her original data, as well as David Shanks, Michael Snodgrass, Uwe Mattler, Dirk Wentura, François Stockart, Liad Mudrik, and many others for helpful discussions of our ideas.
